# Population Pharmacogenomics for Precision Public Health in Colombia

**DOI:** 10.3389/fgene.2019.00241

**Published:** 2019-03-22

**Authors:** Shashwat Deepali Nagar, A. Melissa Moreno, Emily T. Norris, Lavanya Rishishwar, Andrew B. Conley, Kelly L. O’Neal, Sara Vélez-Gómez, Camila Montes-Rodríguez, Wendy V. Jaraba-Álvarez, Isaura Torres, Miguel A. Medina-Rivas, Augusto Valderrama-Aguirre, I. King Jordan, Juan Esteban Gallo

**Affiliations:** ^1^School of Biological Sciences, Georgia Institute of Technology, Atlanta, GA, United States; ^2^IHRC-Georgia Tech Applied Bioinformatics Laboratory, Atlanta, GA, United States; ^3^PanAmerican Bioinformatics Institute, Cali, Colombia; ^4^GenomaCES, Universidad CES, Medellín, Colombia; ^5^Centro de Investigación en Biodiversidad y Hábitat, Universidad Tecnológica del Chocó, Quibdó, Colombia; ^6^Biomedical Research Institute, Cali, Colombia

**Keywords:** pharmacogenomics, pharmacogenetics, precision medicine, genetic ancestry, admixture, Colombia, Antioquia, Chocó

## Abstract

While genomic approaches to precision medicine hold great promise, they remain prohibitively expensive for developing countries. The precision public health paradigm, whereby healthcare decisions are made at the level of populations as opposed to individuals, provides one way for the genomics revolution to directly impact health outcomes in the developing world. Genomic approaches to precision public health require a deep understanding of local population genomics, which is still missing for many developing countries. We are investigating the population genomics of genetic variants that mediate drug response in an effort to inform healthcare decisions in Colombia. Our work focuses on two neighboring populations with distinct ancestry profiles: Antioquia and Chocó. Antioquia has primarily European genetic ancestry followed by Native American and African components, whereas Chocó shows mainly African ancestry with lower levels of Native American and European admixture. We performed a survey of the global distribution of pharmacogenomic variants followed by a more focused study of pharmacogenomic allele frequency differences between the two Colombian populations. Worldwide, we found pharmacogenomic variants to have both unusually high minor allele frequencies and high levels of population differentiation. A number of these pharmacogenomic variants also show anomalous effect allele frequencies within and between the two Colombian populations, and these differences were found to be associated with their distinct genetic ancestry profiles. For example, the C allele of the single nucleotide polymorphism (SNP) rs4149056 [Solute Carrier Organic Anion Transporter Family Member 1B1 (SLCO1B1)^∗^5], which is associated with an increased risk of toxicity to a commonly prescribed statin, is found at relatively high frequency in Antioquia and is associated with European ancestry. In addition to pharmacogenomic alleles related to increased toxicity risk, we also have evidence that alleles related to dosage and metabolism have large frequency differences between the two populations, which are associated with their specific ancestries. Using these findings, we have developed and validated an inexpensive allele-specific PCR assay to test for the presence of such population-enriched pharmacogenomic SNPs in Colombia. These results serve as an example of how population-centered approaches to pharmacogenomics can help to realize the promise of precision medicine in resource-limited settings.

## Introduction

The precision medicine approach to healthcare entails a customized model whereby medical decisions and treatments are specifically tailored to individual patients (Collins and Varmus, 2015; Jameson and Longo, 2015). Currently, precision medicine is most commonly implemented via pharmacogenomic methods, which account for how individuals’ genetic makeup affects their response to drugs (Weinshilboum and Wang, 2006; Ma and Lu, 2011). Pharmacogenomic knowledge of genetic variant-to-drug response interactions provides a means to optimize individual patients’ treatment regimes, simultaneously maximizing drug efficacy while minimizing adverse reactions. Indeed, the essence of precision medicine has been described as “the right treatment, to the right patient, at the right time.” While the precision medicine paradigm promises to revolutionize healthcare delivery, its prohibitive costs put it out of reach for the developing world. In particular, the need to characterize genomic information for each individual patient in a given population can place a tremendous burden on healthcare systems that may be struggling to provide basic services. For the moment, precision medicine as a standard of care is still very much limited to the Global North.

A recently articulated alternative to the precision medicine model is referred to as precision public health (Khoury et al., 2016, 2018; Weeramanthri et al., 2018). The focus of precision public health is populations, instead of individuals, and the idea is to leverage modern healthcare technologies for more precise population-level interventions. The mantra for precision public health is “the right intervention, to the right population, at the right time.” This population-centered model of healthcare delivery provides one way for the technological innovations underlying precision medicine to realize their potential in developing countries. With respect to pharmacogenomics, knowledge regarding population genomic distributions of the genetic variants that mediate drug response can be used to focus resources and efforts where they will be most effective (Bachtiar and Lee, 2013). Under the precision public health model, population genomic profiles, as opposed to genomic information for each individual patient, can be employed to guide pharmacogenomic interventions; this is a far more cost-effective and realistic approach for the developing world (Nordling, 2017). For this study, we applied the precision public health paradigm using a survey of the distribution of pharmacogenomic variants in diverse Colombian populations. The major aim of this work was to tailor pharmacogenomic testing and interventions to the specific populations for which they will realize the greatest benefit.

Colombia is home to a highly diverse, multi-ethnic society. The modern population of Colombia is made up of individuals with genetic ancestry contributions from ancestral source populations in Africa, the Americas, and Europe (Wang et al., 2008; Bryc et al., 2010; Moreno-Estrada et al., 2013; Ruiz-Linares et al., 2014; Rishishwar et al., 2015). Colombia is also known to contain a number of unique regional identities. There are at least five distinct recognized regions in Colombia, each of which has its own defining demographic contours (Appelbaum, 2016; Wade, 2017). In fact, owing to historical barriers to migration, Colombian populations with very different genetic ancestry profiles can be found in close geographic proximity. This is very much the case for the two populations characterized for this study: Antioquia and Chocó (Medina-Rivas et al., 2016; Conley et al., 2017). Despite the fact that these neighboring administrative departments share a common border, their populations show clearly distinct genetic ancestries. Antioquia has primarily European ancestry, whereas Chocó is mainly African, and both populations also show varying levels of Native American admixture.

Previous studies have shown that the frequencies of pharmacogenomic variants can vary across populations with divergent genetic ancestries. This includes variation in pharmacogenomic variant allele frequencies among distantly related populations worldwide (Ramos et al., 2014; Lakiotaki et al., 2017) as well as marked frequency differences among populations sampled from within the same country (Bonifaz-Pena et al., 2014; Hariprakash et al., 2018). We hypothesized that pharmacogenomic allele frequencies should differ between the Colombian populations of Antioquia and Chocó, given their distinct ancestry profiles. If this was indeed the case, it would have direct implications for the development of pharmacogenomic approaches in the country. In this way, we hoped that a survey of the population pharmacogenomic patterns for Antioquia and Chocó could serve as an exemplar for the implementation of precision public health in the developing world.

Colombia’s first clinical genomics laboratory – GenomaCES from Universidad CES in Antioquia^1^ – is currently working to develop genomic diagnoses that are tailored to the local population, and members of the ChocoGen Research Project^2^ are exploring the connections between genetic ancestry and health disparities in the understudied Colombian population of Chocó. Here, these two groups have joined forces in an effort to (i) discover pharmacogenomic variants with special relevance for these two Colombian populations and (ii) develop cost-effective and rapid pharmacogenomic assays for those variants, which can be readily deployed in resource-limited settings.

## Materials and Methods

### Pharmacogenomic SNPs (pharmaSNPs)

Pharmacogenomic single nucleotide polymorphisms (pharmaSNPs), i.e., human genetic variants associated with specific drug responses, were mined from the Pharmacogenomic Knowledgebase (PharmGKB^3^) (Whirl-Carrillo et al., 2012). PharmGKB provides a manually curated set of clinical annotations with information about pharmaSNPs and their corresponding drug responses. The PharmGKB clinical annotations were downloaded and filtered to extract all individual pharmaSNP clinical annotations. Data on pharmaSNP clinical annotations were parsed and stored, including information about the direction and nature of the variant associated drug responses, the identity of each pharmaSNP effect and non-effect allele, the genes wherein pharmaSNPs are located, and the drug interaction evidence levels.

### PharmaSNP Genetic Variation

Data on human genome sequence variation were taken from the phase 3 data release of the 1000 Genomes Project (Genomes Project et al., 2015). For the 1000 Genomes Project, genome-wide SNPs were characterized via whole genome sequencing for 2504 individuals from 26 global populations, including the Colombian population of Antioquia [Colombian in Medellín (CLM), Colombia^4^]. All of the pharmaSNPs from PharmGKB were found to be present in 1000 Genomes Project phase 3 variant calls. Genome sequence variation for the Colombian population of Chocó was characterized as part of the ChocoGen Research Project^5^ as previously described (Medina-Rivas et al., 2016; Chande et al., 2017; Conley et al., 2017).

Genome sequence variation data were used to calculate the average minor allele frequency (MAF) and fixation index (F_ST_) for a genome-wide set of *n* = 28,137,656 pruned SNPs and for the set of *n* = 1995 pharmaSNPs using the program PLINK (Purcell et al., 2007). Linkage disequilibrium pruning was performed to yield the genome-wide background SNP set with the PLINK indep command, using an *r*^2^ threshold of 0.5 with a sliding window of 50 nt and a step size of 5 nt. MAF (p) values for each SNP were calculated across all populations as: $p=\frac{\text{number of variant sites}}{\text{total number of sites}}$. F_ST_ values for each SNP were calculated among populations as: $F_{ST}=\frac{\sigma^{2}}{\overset{¯}{p}\times \left(1-\overset{¯}{p}\right)}$, where $\overset{¯}{p}$ is the average MAF across all 26 global populations and *σ*^2^ is the observed MAF variation. Pairwise genomic distances were computed as 1*-identity-by-state/Hamming distances* between genomes using the PLINK distance command with the –distance-matrix option. The resulting high-dimensional pairwise genomic distance matrix was projected in two dimensions using multi-dimensional scaling (MDS) method implemented in the base package of the R statistical language (R Core Team, 2013). The program ADMIXTURE was used to characterized genetic ancestry components based on the genome-wide and pharmaSNP sets using *K* = 3 clusters (Alexander et al., 2009).

The differences in pharmaSNP effect allele frequencies (*f*) between Antioquia (*ANT*) and Chocó (*CHO*) were measured as (1) the log-transformed ratio of the population-specific allele frequencies log_2_(*f*_*ANT*_/*f*_*CHO*_) and (2) as the population-specific allele frequency difference Δ = *f*_*ANT*_/*f*_*CHO*_. These two effect allele difference metrics were plotted orthogonally and the Euclidean distance from the origin was calculated for each pharmaSNP to yield a composite difference.

### PharmaSNP Ancestry Associations

The influence of genetic ancestry on pharmaSNP genotype frequencies was measured via ancestry association analysis. To do this, individuals’ genetic ancestry fractions – African, European, and Native American – inferred using ADMIXTURE with the genome-wide SNP set, were regressed against their individual pharmaSNP genotypes. The strength of the resulting ancestry × pharmaSNP associations were quantified using a linear regression model: *y = βx + ε*, where x ∈ {0, 1, 2}, corresponding to the number of pharmaSNP effect alleles, *y* is the ancestry fraction for a given ancestral group (African, European, or Native American), and β quantifies the strength of the association. The significance of the ancestry association is measured as the *P*-value obtained from a *t*-test, where *t = β/SE_β_*.

### Exome Sequence Analysis

Whole exome sequence (WES) analysis was conducted on a cohort of 132 de-identified patients characterized for the purposes of genetic testing by the GenomaCES laboratory (O’Donnell-Luria and Miller, 2016). The study was carried out in accordance with article 11 of resolution 8430 of 1993 of Colombian law, which states that for every investigation in which a human being is the study subject, respect for their dignity and the protection for their rights should always be present. The study protocol was reviewed and approved by the ethics committee and the research committee of Universidad CES, and all subjects gave written informed consent authorizing use of their biological samples and genetic information obtained through exome sequencing for research and academic training in accordance with the Declaration of Helsinki. Patient DNA was extracted from peripheral blood using the salting out method (Miller et al., 1988). Exon enrichment was performed using the Integrated DNA Technologies xGen capture kit, and exome sequencing was performed on the Illumina HiSeq 4000, generating 150 bp paired end reads at 100X coverage. Read quality was assessed using the FastQC program with a threshold of *Q* ≥ 30 (Andrews, 2010). Sequence reads were mapped to the hs37d5 (1000 Genomes Phase II) human genome reference sequence using SAMtools (Li et al., 2009), and variants were called using VarScan 2 (Koboldt et al., 2012). The resulting VCF files were surveyed for the presence of pharmaSNP alleles using the VCFtools package (Danecek et al., 2011). Manual inspection of the mapped sequence reads in support of pharmaSNP variant calls was performed using the Integrative Genomics Viewer (IGV) (Thorvaldsdottir et al., 2013).

### Allele-Specific PCR Assay

The identity of pharmaSNP allelic variants was assayed in the same 132 patients using custom-designed allele-specific PCR assays following the Web-based Allele-Specific PCR (WASP) primer design protocol (Wangkumhang et al., 2007). Both the WASP and Primer-BLAST (Ye et al., 2012) tools were used to design pairs of allele-specific forward primers that overlap with the pharmaSNPs of interest and their corresponding single reverse primers. PCR assays were performed using the Thermo Scientific^TM^ Taq DNA Polymerase kit, with 25 μL final reagent volume, on the Bio-Rad thermocycler (C1000 Touch^TM^ Thermal Cycler). PCR products were visualized and scored as homozygous non-effect allele, heterozygous, or homozygous effect allele using electrophoresis performed with 2.5% agarose gels stained with ethidium bromide (10 μL) with a running time of 60 min at 70 V in 1X TBE buffer. UV light was used to visualize the gel-separated PCR products.

## Results

### Pharmacogenomic SNP Variation Worldwide

We operationally define pharmaSNPs as human nucleotide variants that are known to affect how individuals respond to medications. The Pharmacogenomics Knowledgebase (PharmGKB^6^) provides a catalog of pharmaSNPs together with information regarding their known impacts on drug response. PharmGKB categorizes pharmaSNPs with respect to their specific effects on drug efficacy, dosage, or toxicity/adverse drug reactions as well as the level of evidence for their role in drug response: (1) high, (2) moderate, (3) low, or (4) preliminary. We mined the PharmGKB database for pharmaSNPs across all four evidence levels, yielding a total of 1995 SNPs genome-wide.

**FIGURE 1:**
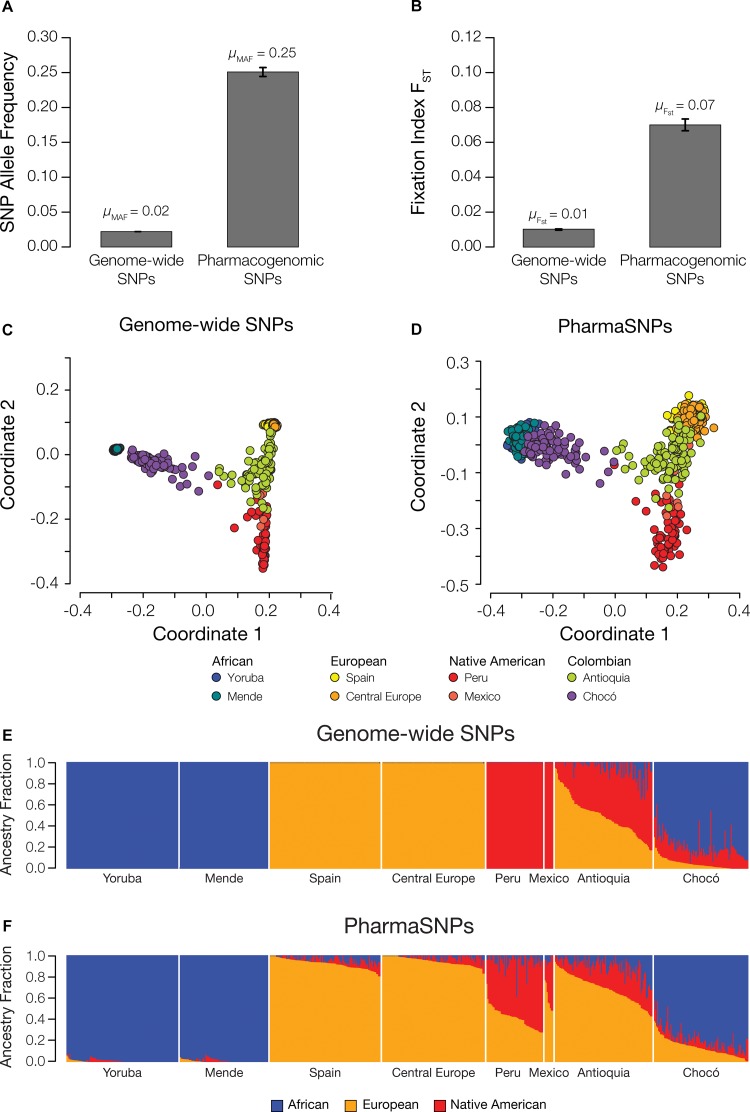
Patterns of variation for pharmacogenomic SNPs (pharmaSNPs) worldwide. Average **(A)** minor allele frequency (MAF) and **(B)** fixation index (F_ST_) values for all genome-wide SNPs (*n* = 28,137,656) and all pharmaSNPs (*n* = 1995) across the 26 1KGP populations studied here. Multi-dimensional scaling (MDS) plots showing the inter-individual genetic distances of admixed Colombian individuals (Antioquia and Chocó) in relation to global reference populations from Africa, Europe, and the Americas for **(C)** genome-wide SNPs and **(D)** pharmaSNPs. ADMIXTURE plots showing the genome-wide continental ancestry fractions using **(E)** all genome-wide SNPs and **(F)** only pharmaSNPs for admixed Colombian populations (Antioquia and Chocó) and reference African (blue), European (orange), and Native American (red) populations.

We evaluated the global patterns of pharmaSNP variation using whole genome sequence data for 26 populations from five continental (super) population groups characterized as part of the 1000 Genomes Project (Genomes Project et al., 2015). Levels and patterns of variation for pharmaSNPs were compared to a genome-wide background set of >28 million SNPs. Across all 26 global populations, pharmaSNPs show a very high average MAF (avg. MAF = 0.25) compared to genome-wide SNPs (avg. MAF = 0.02; Figure 1A). PharmaSNPs also show significantly higher levels of the fixation index (avg. F_ST_ = 0.07), a measure of between-population differentiation, for global populations compared to genome-wide SNPs (avg. F_ST_ = 0.01; Figure 1B). It should be noted that the higher avg. MAF observed for pharmaSNPs compared to genome-wide SNPs could reflect an ascertainment bias owing to a relative excess of rare variants in the 1000 Genomes Project sequence data. However, no such bias is expected for the F_ST_ values as calculated here, which are largely unaffected by the presence of rare variants in the 1000 Genomes Project data (Bhatia et al., 2013).

Given the high levels of variation and between-population discrimination shown by pharmaSNPs, we also evaluated the extent to which they carry information about genetic ancestry and admixture, particularly for the Colombian populations of Antioquia and Chocó. Pairwise genomic distances were computed for the Colombian populations together with a set of global reference populations from Africa, the Americas, and Europe, using both pharmaSNPs and the genome-wide SNP set. Pairwise genomic distances computed using both sets of SNPs were used to reconstruct the evolutionary relationships among human populations worldwide. The results for the genome-wide (Figure 1C) and pharmaSNP (Figure 1D) sets are highly similar. The genome-wide SNP set does provide higher resolution and tighter groupings than the pharmaSNPs, but the nature of the relationships among global populations does not change between the two SNP sets. The African, European, and Native American populations occupy the three poles of the MDS plot, with Antioquia falling along the axis between the European and Native American groups and Chocó grouping more closely with the African populations. Both Colombian populations show evidence of substantial admixture compared to the global reference populations.

We performed a similar comparison of the ability pharmaSNPs to quantify patterns of genetic ancestry compared to genome-wide SNPs using the program ADMIXTURE. Using *K* = 3 ancestry components, genome-wide SNPs clearly distinguish the reference African, European, and Native American populations, and characterize the Colombian populations of Antioquia and Chocó as distinct mixtures of all three ancestries (Figure 1E). Consistent with previous results (Conley et al., 2017), Antioquia shows an average of 61% European, 32% Native American, and 7% African ancestry, whereas Chocó shows primarily African ancestry (76%) followed by 13% Native American, and 11% European fractions. PharmaSNPs show qualitatively similar results albeit with lower resolution compared to the genome-wide SNP set (Figure 1F). Using pharmaSNPs, the global reference populations are not quite as distinct and the European component of ancestry appears to be overestimated in both the Native American reference populations as well as Antioquia and Chocó. Nevertheless, the clear distinction between the patterns of ancestry and admixture for the Colombian populations, whereby Antioquia is primarily European and Chocó is mostly African, is captured when only the pharmaSNPs are used.

### Pharmacogenomic SNP Variation in Colombia: Antioquia Versus Chocó

**FIGURE 2:**
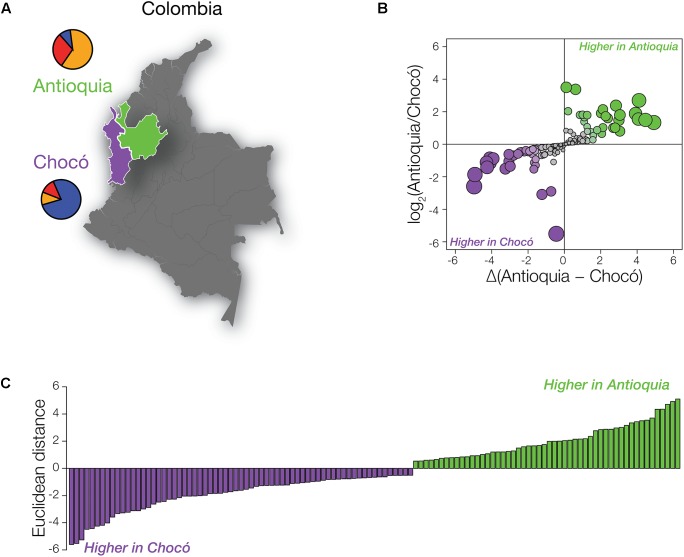
PharmaSNPs with population-specific effect allele frequency differences in Colombia. **(A)** Map of Colombia, highlighting Antioquia in green and Chocó in purple. Population-specific mean ancestry fractions are shown as pie charts: African (blue), European (orange), and Native American (red). **(B)** Comparison of the ratio of pharmaSNP effect allele frequency differences between Antioquia and Choco (*y*-axis) to the magnitude of the frequency differences (*x*-axis). Circles are scaled according to their Euclidean distance (distance from the origin) and are colored to indicate the direction of their difference (green – higher effect allele frequency in Antioquia; purple – higher effect allele frequency in Chocó). **(C)** Distribution of pharmaSNPs with Euclidean distance > 0.5. Green indicates that the pharmaSNP effect allele is more frequent in Antioquia, while purple indicates that the effect allele is more frequent in Chocó.

Despite the fact that the Colombian administrative departments of Antioquia and Chocó are located in close proximity, their populations have distinct global origins (Figure 2A). As discussed in the previous section and elsewhere (Rishishwar et al., 2015; Medina-Rivas et al., 2016; Conley et al., 2017), the population of Antioquia shows mainly European genetic ancestry with substantial Native American admixture, whereas Chocó has primarily African ancestry with lower levels of Native American and European admixture. In light of the high levels of global variation seen for pharmaSNPs (Figure 1), we expected to see pronounced differences in the distributions of pharmaSNP alleles between Antioquia and Chocó. Such differences should have implications for public health strategies in the country, particularly with respect to the allocation of resources for pharmacogenomic testing.

We compared the frequencies of pharmaSNP effect alleles between Antioquia and Chocó to test this hypothesis. PharmaSNP effect alleles are operationally defined for this purpose as the allelic variants that increase the observed effect for a given drug-gene interaction, i.e., the alleles that increase the efficacy, dosage, or risk of toxicity/adverse drug responses for a drug. To ensure maximum relevance of our results for public health in Colombia, we focused on pharmaSNPs corresponding to the highest evidence levels in PharmGKB (levels 1 and 2; *n* = 155 pharmaSNPs). PharmaSNP effect allele frequency differences between Antioquia and Chocó were measured in two ways – (1) as the log transformed ratio of allele frequencies Antioquia/Chocó and (2) as the allele frequency differences between Antioquia and Chocó – in order to capture both high relative differences at low allele frequencies and high absolute differences at high allele frequencies (Figure 2B). When these two dimensions of pharmaSNP effect allele frequency differences are plotted orthogonally, the Euclidean distance from the origin captures the overall between-population difference seen for each SNP (Figure 2C).

**FIGURE 3:**
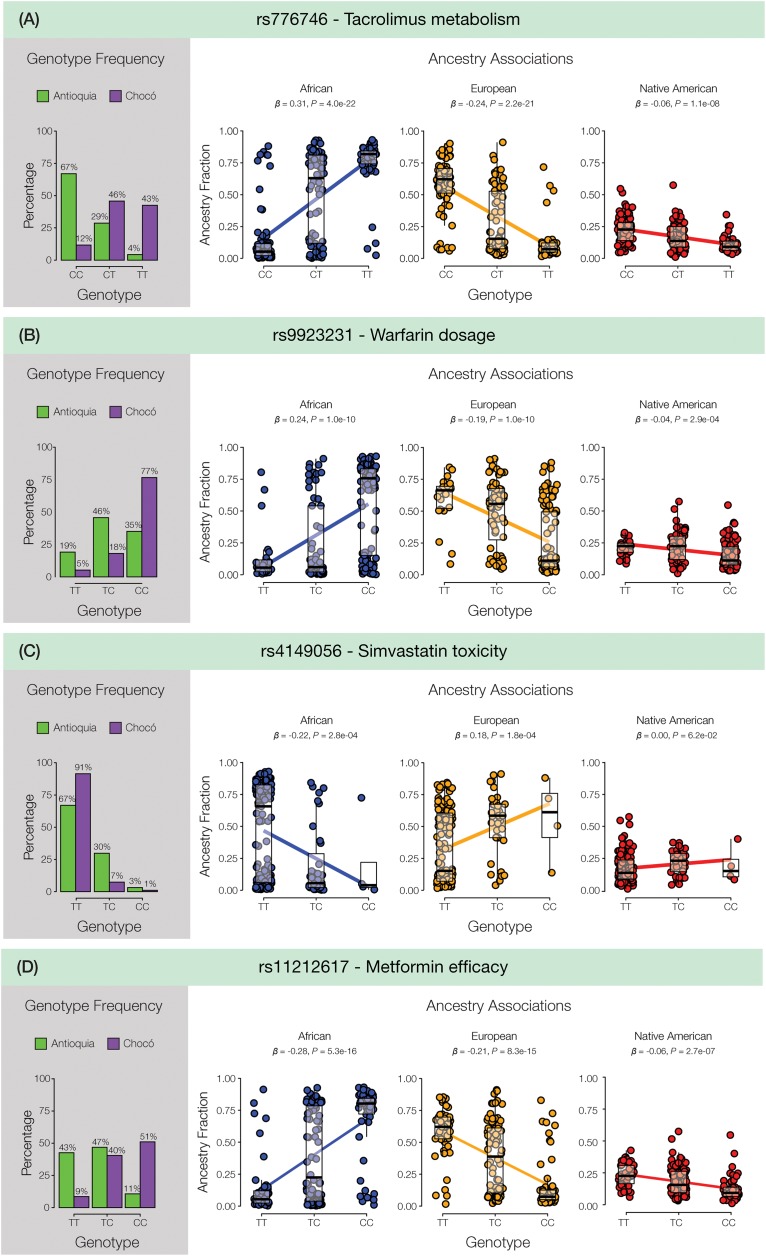
Ancestry associations for pharmaSNPs in Colombia. For each panel in the figure, pharmaSNP genotype frequencies are shown for Antioquia (green) and Chocó (purple) followed by the ancestry association plots. For each genetic ancestry component – African (blue), European (orange), and Native American (red) – individuals’ ancestry fractions (*y*-axis) are regressed against their pharmaSNP genotypes (*x*-axis). Ancestry associations are quantified by the slope of the regression (β) and its significance level (*P*). Results are shown for **(A)** the tacrolimus metabolism-associated SNP rs776746 (CYP3A5^∗^3), **(B)** the warfarin dosage-associated SNP rs9923231 (VKORC1^∗^2), **(C)** the simvastatin toxicity-associated SNP rs4149056 (SLCO1B1^∗^5), and **(D)** the metformin efficacy-associated SNP rs11212617.

As expected, numerous pharmaSNP effect alleles show large frequency differences between Antioquia and Chocó (Figure 2). We sought to quantify the role that the distinct genetic ancestry profiles of these two populations plays in these pharmaSNPs effect allele frequency differences. To do so, we developed and applied an ancestry association method whereby individuals’ genetic ancestry fractions – African, European, and Native American – are regressed against their genotypes for any given pharmaSNP. This approach allows us to visualize and quantify the influence of genetic ancestry on pharmaSNPs genotype frequencies in these two diverse Colombian populations. Figure 3 shows examples of ancestry associations for three pharmaSNPs with high levels of effect allele (and genotype) divergence between Antioquia and Chocó; ancestry associations for nine additional pharmaSNPs of interest to Colombia can be seen in Supplementary Figure S1. Table 1 shows the results of ancestry association analyses for 13 pharmaSNPs of interest to Colombia, based on high levels of divergence between Antioquia and Chocó, and Supplementary Table S1 contains the ancestry association results for all level 1 and 2 PharmGKB SNPs showing pharmaSNP effect allele Euclidean distances > 0.5 (as shown in Figure 2C).

**Table 1:** Colombian ancestry-associated pharmaSNPs of interest.

PharmaSNP	Effect	Effect allele frequencies		African ancestry correlation		European ancestry correlation		Native American ancestry correlation	
					
		Antioquia	Chocó	β value	*P*-value	β value	*P*-value	β value	*P*-value
rs776746^∗^	Tacrolimus metabolism	0.81	0.32	0.31	4.00e-22	-0.24	2.20e-21	-0.06	1.10e-08
rs1799853	Warfarin dosage	0.88	0.97	-0.25	4.20e-04	0.21	2.50e-04	0.04	8.40e-02
rs9923231^∗^	Warfarin dosage	0.57	0.88	0.24	1.00e-10	-0.19	1.00e-10	-0.04	2.90e-04
rs4149056^∗^	Simvastatin toxicity	0.18	0.05	-0.22	2.80e-04	0.18	1.80e-04	0.00	6.20e-02
rs4244285	Clopidogrel efficacy	0.90	0.84	0.09	1.36e-01	-0.06	1.87e-01	-0.02	1.67e-01
rs2740574	Tacrolimus metabolism	0.10	0.59	0.32	1.20e-22	-0.24	2.20e-21	-0.06	1.00e-09
rs11615	Platin toxicity	0.48	0.07	-0.30	9.80e-18	0.25	2.50e-19	0.04	1.10e-04
rs11212617	Metformin efficacy	0.33	0.74	0.28	5.30e-16	-0.21	8.30e-15	-0.06	2.70e-07
rs6977820	Antipsychotic drug toxicity	0.25	0.68	0.28	5.30e-16	-0.19	1.70e-12	-0.07	1.20e-11
rs3812718	Antiepileptic treatment resistance	0.55	0.27	-0.27	2.00e-06	0.21	8.40e-06	0.05	7.74e-04
rs7793837	Salbutamol efficacy	0.69	0.24	0.21	4.30e-17	0.21	2.90e-16	0.05	2.80e-07
rs1954787	Antidepressant efficacy	0.62	0.21	-0.24	3.00e-13	0.18	6.80e-12	0.05	4.20e-07
rs1719247	Simvastatin adverse reaction	0.54	0.27	-0.21	3.90e-08	0.17	3.50e-08	0.04	2.00e-03


*The pharmaSNPs marked with an asterisk (^∗^) are shown in Figure 3.*

#### Tacrolimus

The T allele of the pharmaSNP rs776746 (CYP3A5^∗^3) is found at higher frequency in Chocó and is positively correlated with African ancestry and negatively correlated with both European and Native American ancestry (Figure 3A). This pharmaSNP is a splice site acceptor variant located within an intron of the CYP3A5 (Cytochrome P450 Family 3 Subfamily A Member 5) encoding gene. The T allele is associated with increased metabolism of Tacrolimus, an immunosuppressive drug often used to treat transplant patients, and thus individuals with T containing genotypes may require relatively higher dosages of this drug. Consistent with these observations, physicians in Cali, Colombia, have anecdotally reported that Afro-Colombian transplant patients do not respond well to standard doses of Tacrolimus.

#### Warfarin

The C allele of the pharmaSNP rs9923231 (VKORC1^∗^2) shows a similar pattern with higher frequency in Chocó, a positive correlation with African ancestry, and negative correlations with both European and Native American ancestry (Figure 3B). This pharmaSNP is one of several variants of the VKORC1 (Vitamin K Epoxide Reductase Complex Subunit 1) encoding gene that have been associated with warfarin sensitivity. The SNP is located in the upstream, regulatory region of the gene, and individuals with the C allele may require an increased dosage of warfarin.

#### Simvastatin

The C allele of the pharmaSNP rs4149056 (SLCO1B1^∗^5) is found in higher frequency in Antioquia, showing a negative correlation with African ancestry and a positive correlation with European ancestry (Figure 3C). The correlation with Native American ancestry for this SNP is not significant. This SNP is a missense variant in the SLCO1B1 encoding gene. The C allele is associated with simvastatin toxicity, and individuals with this allele may be at higher risk for simvastatin-related myopathy. These results agree very well with observations of physicians from the Universidad CES clinic in Antioquia, who have observed that ∼30% of patients treated with Simvastatin show evidence of adverse drug reactions.

#### Metformin

The C allele of the pharmaSNP rs11212617 is found at substantially higher frequency in Chocó compared to Antioquia, and it is positively correlated with African ancestry and negatively correlated with both European and Native American ancestry (Figure 3D). This pharmaSNP shows an interaction with the type 2 diabetes drug Metformin; the C effect allele was found to be associated with greater treatment success (GoDARTS and Ukpds Diabetes Pharmacogenetics Study Group et al., 2011). Interestingly, metformin was subsequently proven to have higher efficacy for the reduction of blood glucose levels reduction in African–Americans compared to European–Americans (Williams et al., 2014; Zhang and Zhang, 2015). Ergo, this ancestry-associated pharmaSNP shows a direct connection between genetic ancestry differences and differential drug response.

### Cost-Effective pharmaSNP Genotyping in Colombia With Allele-Specific PCR

The results from the analysis of pharmaSNP variation in Colombia uncovered a number of SNPs with specific relevance to the country, in terms of anomalous effect allele frequencies within local populations, associations with different genetic ancestry groups, and broad relevance to public health. We reasoned that such population genomic profiling can be used to focus efforts to develop precision medicine in the country and to maximize the return on investment for pharmacogenomic testing in resource-limited settings. To this end, GenomaCES developed and validated three custom allele-specific PCR assays to genotype pharmaSNPs of special relevance to these Colombian populations.

The criteria for the selection of pharmaSNPs that were interrogated with our custom allele-specific PCR assays included the PharmGKB evidence level along with a combination of population genomic and clinical information. Pharmacogenomic assays were only developed for pharmaSNPs from the PharmGKB evidence level 1A. This is the highest evidence level and corresponds to pharmaSNPs that are included in medical society-endorsed pharmacogenomics guidelines and/or implemented in major health systems. The additional criteria used to prioritize pharmaSNPs for the development of allele-specific PCR assays were: (i) observations of population-specific allele frequencies in Colombia along with related ancestry-associations, (ii) pharmacogenomic associations with drugs that are widely prescribed in Colombia and used to treat common conditions, and (iii) pharmacogenomic associations with drugs for which GenomaCES investigators have anecdotal information from collaborating physicians that pharmacogenomic tests would be of use to the local population, based on their observations of anomalous drug responses in their patients. It should be noted that the population and clinical criteria are not mutually exclusive; indeed, physicians’ observations of anomalous drug responses in their patient populations are almost certainly related to the population-specific allele frequencies of the relevant pharmaSNPs.

**FIGURE 4:**
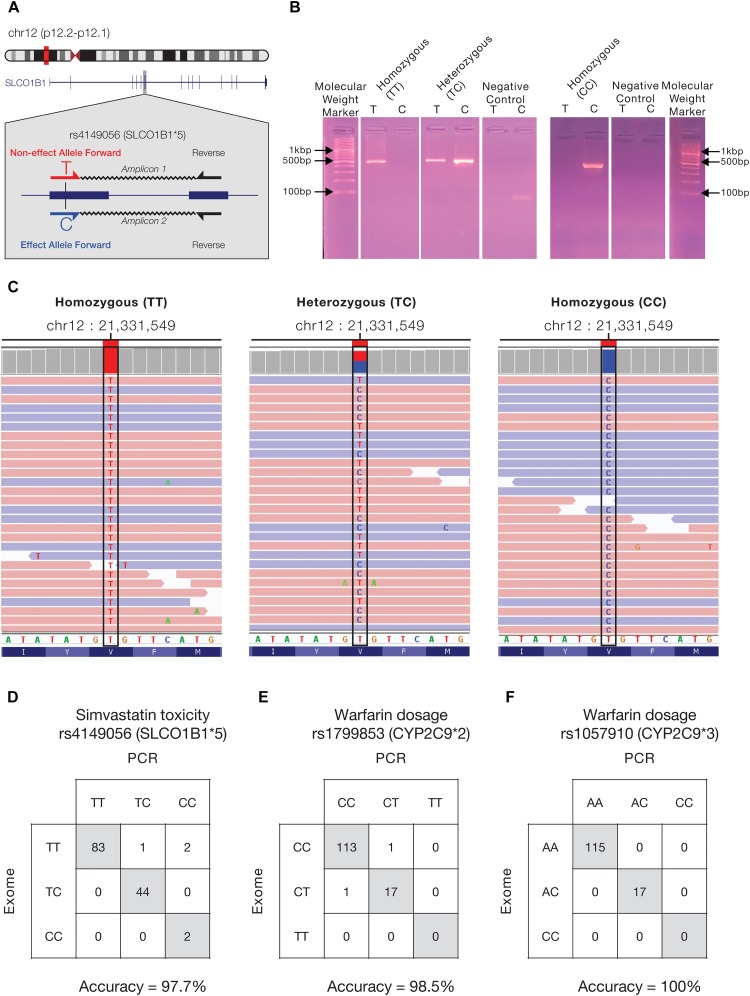
Allele-specific PCR assay for pharmaSNPs. **(A)** Schema depicting the design of the allele-specific PCR assay for the pharmaSNP rs4149056 (SLCO1B1^∗^5) on chromosome 12. Two allele-specific forward primers are designed for the pharmaSNP of interest and paired with a single reverse primer, yielding allele-specific amplicons. **(B)** Allele-specific PCR results for four individuals are shown. PCR gel lanes are labeled with the allele used for the forward primer – T or C. **(C)** Results of exome sequence analysis used to confirm the results of the allele-specific PCR assays. Sequence reads (red – forward, blue – reverse) mapped to the genomic position for the SNP rs4149056, coverage levels (gray boxes above), and the identity of the called nucleotide variants at that same position are shown along with the reference nucleotide and amino acid sequences for the corresponding region of the *SLCO1B1* gene (protein). Images were taken from the Integrative Genomics Viewer (IGV). Confusion matrices showing comparisons between the pharmaSNP variant calls made via exome sequence analysis and the allele-specific PCR assays are shown for **(D)** the simvastatin toxicity SNP rs4149056 (SLCO1B1^∗^5), and the warfarin dosage SNPs **(E)** rs1799853 (CYP2C9^∗^2) and **(F)** rs1057910 (CYP2C9^∗^3). Identical variant calls are shown along the diagonal, whereas off-diagonal calls show discrepancies between the exome and PCR variant calls; accuracy levels for each test are shown.

An example of an allele-specific PCR assay developed for the simvastatin-associated pharmaSNP rs4149056 (SLCO1B1^∗^5), located with an exon of the SLCO1B1 protein coding gene on the short arm of chromosome 12, is shown in Figure 4A. The pharmaSNP variant detection assay relies on the use of two forward primers – one to capture the non-effect allele T and one to capture the effect allele C – and a single reverse primer. Use of these two primer-pairs results in allele-specific amplicons, depending on the presence of each allele in an individual patient’s genome. PCR results are shown for four patients: Patient-132 homozygous TT, Patient-44 heterozygous TC, and Patient-17 and Patient-26 homozygous CC (Figure 4B). We visualized the results of exome sequence analysis, with respect to the quality and coverage of mapped reads along with the counts of the different variant calls, to manually confirm the results of the allele-specific PCR assays (Figure 4C).

Having confirmed the accuracy of the rs4149056 (SLCO1B1^∗^5) variant detection assay, we then ran it on a cohort of 132 de-identified patients from the GenomaCES laboratory, all of whom have exome sequences available for confirmatory analysis. The results of the allele-specific PCR and exome analyses are highly similar; taking the exome results as the ground truth against which to compare the PCR assay yields an overall accuracy of 97.7% for this test (Figure 4D). Two additional allele-specific PCR assays for SNPs associated with warfarin dosage – rs1799853 (CYP2C9^∗^2) and rs1057910 (CYP2C9^∗^3) – were tested on the same patient set and confirmed via exome sequence analysis. These two allele-specific PCR genotyping assays show even higher accuracies of 98.5 and 100%, respectively. We calculated a number of additional performance metrics for all three of these tests, breaking down each assay into its three constituent genotypes, the results of which are shown in Supplementary Figure S2.

## Discussion

### Caveats and Limitations

We would like to point out some of the caveats and limitations of the current study as they relate to the accuracy and utility of pharmacogenomic tests in understudied populations. The reach of our analysis is somewhat limited by the focus on pharmaSNPs, i.e., single nucleotide variants, as opposed to all possible genetic variants that may impact drug response. PharmGKB contains annotations of gene-to-drug response interactions that are mediated by a number of different kinds of variants, including larger scale structure variants such as insertion/deletion events and copy number variations (Roden et al., 2006; He et al., 2011). Furthermore, there are a number of pharmacogenomic tests that rely on the characterization of combinations of linked SNPs, i.e., haplotypes or star-alleles. For example, the most reliable warfarin sensitivity assays utilize multiple SNPs (haplotypes) across two genes in order to arrive at specific dosage recommendations (Johnson et al., 2011; Fung et al., 2012). Our survey of pharmaSNP variation will not capture these complex classes of pharmacogenomic variants and interactions.

Our focus on pharmaSNPs can be primarily attributed to the availability and the reliability of SNP data at our disposal, as opposed to other more complex genetic variants, particularly for the population of Chocó, which was characterized using a genome-wide SNP array (Medina-Rivas et al., 2016; Chande et al., 2017; Conley et al., 2017). Nevertheless, it is important to note that (i) there are numerous documented cases of individual SNPs that show demonstrable and reproducible effects on drug response (Lauschke et al., 2017) and (ii) there are many more pharmaSNPs available for analysis compared to the other variant classes (Whirl-Carrillo et al., 2012). For example, ∼93% of PharmGKB variant annotations correspond to individual pharmaSNPs (1995 out of 2144 total variants). Accordingly, we are confident that our study design captures the majority of the pharmacogenomically relevant human genetic variation based on current knowledge in the field.

Another limitation relates to the fact that we compared pharmaSNP allele frequencies among populations with distinct ancestries compared to the cohorts where they were originally characterized. As with other classes of clinical genetics studies (Petrovski and Goldstein, 2016; Popejoy and Fullerton, 2016), there remains a very strong bias whereby the majority of pharmacogenetic clinical trials have been conducted in developed countries on cohorts with European ancestry (Karlberg, 2008; Thiers et al., 2008). Thus, it is formally possible that the pharmaSNPs we analyzed may have different effects on drug response in our populations of interest. Of course, the most rigorous way to assess the population-specific role of genetic variation in drug response would be to conduct clinical trials in all populations of interest. Currently, however, the high cost and complexity of performing clinical trials across multiple populations, particularly for variants with already well documented effects on drug response, renders this approach prohibitive. In addition, it is important to point out that the associations between pharmaSNPs and drug response that our study relies on are far more likely to be causal than associations uncovered by genome-wide association studies (GWAS), many of which do not replicate across populations with distinct ancestry profiles (Martin et al., 2017). This is because GWAS SNPs do not correspond to causal variants *per se*; rather, they are tag variants that mark haplotypes wherein the causal SNPs lie, and haplotype structure is known to vary widely across populations (Conrad et al., 2006). PharmaSNPs, on the other hand, correspond to the specific causal variants for which there is direct evidence of an impact on drug response. This is particularly the case for the narrower set of 155 pharmaSNPs deemed to be most confident by PharmGKB, which we used for our comparison of Antioquia and Chocó. The strong clinical and experimental evidence of these high confidence pharmaSNPs effects on drug response gives us confidence with respect to their potential relevance for our populations of interest.

### The Underlying Complexity of So-Called Hispanic/Latino Populations

As briefly mentioned in the previous section, a number of recent studies have underscored the major sampling bias that currently exists for human clinical genomic studies and emphasized the corollary importance of extending clinical trials to currently understudied populations. These studies rely on a variety of labels related to “Hispanic/Latino” to describe understudied populations from Latin America, or individuals and communities with origins in Latin America. For example, in a survey of the ancestry of study participants in GWAS cohorts, the authors used the label “Hispanic and Latin American ancestry,” showing that members of this group made up a mere 0.06% of GWAS study participants in 2009 and 0.54% in 2016 (Popejoy and Fullerton, 2016). Another study, which demonstrated the importance of using matched ancestry samples for clinical variant interpretation, employed the category “Latino ethnicity” to classify exome variants into a single control group (Petrovski and Goldstein, 2016). The widely used Exome Aggregation Consortium (ExAC) database uses the term “Latino” as a population category for exome sequence variants (Lek et al., 2016), and the 1000 Genomes Project uses the super population code “Ad Mixed American (AMR)” to group genetically diverse populations from Colombia, Mexico, Peru, and Puerto Rico (Genomes Project et al., 2015).

It is interesting to note that the origins of the term Hispanic/Latino as a catch-all phrase to describe an extraordinarily diverse set of populations can be traced to decisions imposed by activists and bureaucrats of the US Census Bureau, motivated by the opportunity to create a politically influential interest group (Mora, 2014). The results of our study highlight the artificial nature, and the lack of practical utility, of the Hispanic/Latino label as it pertains to clinical genetic studies. Our two populations of interest – Antioquia and Chocó – would both be considered Hispanic/Latino, and in fact they are both from the same country within Latin America, but they have very distinct patterns of genetic ancestry and admixture. Furthermore, we show here that the differences in genetic ancestry have specific implications for the pharmacogenomic profiles of each population. The same thing will certainly hold true for many other sets of populations both within and between different Latin American countries. In light of this realization, we would like to emphasize that the stratification of so-called Hispanic/Latino populations for clinical genetic studies should be performed using their distinct genetic ancestry profiles as opposed to a politically imposed pan-ethnic label.

### Population-Guided Approaches to Pharmacogenomics in the Developing World

We hope that the population pharmacogenomic approach we applied to Colombian populations in this study can serve as model for their broader application in the developing world. Currently, genomic approaches to precision medicine are prohibitively expensive for many developing countries owing to their reliance on deep genetic characterization of individual patients. Precision public health, on the other hand, entails population-level interventions, and the focus on populations can provide a more cost-effective means for the implementation of novel genomic approaches to healthcare (Khoury et al., 2016; Khoury et al., 2018; Weeramanthri et al., 2018). Population-guided approaches to pharmacogenomics allow healthcare providers to allocate resources and efforts where they will be most effective by uncovering pharmacogenomic variants with special relevance to specific populations (Bachtiar and Lee, 2013; Nordling, 2017).

Here, we report a number of examples of pharmacogenomic variants with anomalously high effect allele frequencies in distinct Colombian populations. For example, the T allele of the pharmaSNP rs776746 is associated with African ancestry and found at a relatively high frequency in Chocó (Figure 4 and Table 1). Since this variant is associated with the need for a higher dosage of the immunosuppressive drug Tacrolimus, Afro-Colombians may be particularly prone to organ rejection following allogeneic transplant. Accordingly, the local deployment of a pharmacogenomic test for this particular SNP in Chocó would simultaneously focus limited resources for genetic testing while also ensuring an outsized impact for Afro-Colombian patients. As another example, the population of Antioquia shows an elevated frequency of the C allele of the pharmaSNP rs4149056, which is associated with increased risk of simvastatin toxicity (Figure 4 and Table 1). The development of a pharmacogenomic assay for this SNP, which is currently underway at GenomaCES in Antioquia, could help to mitigate the risk of adverse drug reactions to this commonly prescribed medication in the local population.

### Prospects for Pharmacogenomics in Colombia

This is an auspicious moment for the development of pharmacogenomic approaches to public health in Colombia. The Colombian biomedical community is simultaneously faced with a combination of great opportunities and profound challenges, both with respect to genomic medicine overall and for pharmacogenomics in particular (De Castro and Restrepo, 2015). In all of South America, Colombia is one of only two countries, together with Argentina, with nationalized healthcare systems that guarantee comprehensive coverage for all of its citizens. In 2015, the terms of this guarantee were updated, via the Ministry of Health and Social Protection resolution 5592, to cover broadly defined molecular genetic and genomic tests. This change resulted in a far more comprehensive coverage policy for these kinds of tests than currently exists in the United States, where many precision medicine treatments are still directly paid by patients (Szabo, 2018). This resolution reflects great foresight on the part of Colombian policy makers and represents a tremendous opportunity for local biomedical researchers, clinicians, and the patients that they serve. Furthermore, a very strong case has been made for how genome-enabled approaches to precision medicine should ultimately lead to substantial cost savings for the national healthcare system over the long term (Gallo, 2017; Gibson, 2018).

On the other hand, the costs of many of the tests covered by this policy are so expensive in Colombia that the sustainability of the policy has been called into serious question. For example, the molecular biology reagents needed for tests of this kind can often cost three times as much or more in Colombia, compared to the United States, owing to taxes and tariffs. We firmly believe that key solutions to this economic challenge will be to (i) build the local capacity needed to perform such tests and (ii) develop genomic assays that are specifically tailored to the needs of Colombian populations. To these ends, Universidad CES has invested substantially in the development of local capacity in genomic medicine via the establishment of GenomaCES, which is Colombia’s first homegrown genomic medicine laboratory. As we have shown here, GenomaCES is working to develop inexpensive and rapid pharmacogenetic genotyping tests based on relatively simple allele-specific PCR assays. Developing local tests of this kind can help to ensure that variants of specific relevance to the country are prioritized for testing and to avoid the prohibitively high costs of commercially available tests and/or kits.

## Data Availability

All datasets generated for this study are included in the manuscript and/or the Supplementary Files.

## Author Contributions

AV-A, IJ, and JG conceived of and designed the study. SN, AM, EN, LR, AC, KO’N, SV-G, and CM-R performed all data analysis. AM, SV-G, WJ-Á, and IT performed laboratory assays. MM-R, AV-A, IJ, and JG supervised and managed all aspects of the project in Colombia and the United States. MM-R and JG acquired study subject samples. SN, LR, and IJ prepared figures and wrote the manuscript.

## Conflict of Interest Statement

The authors declare that the research was conducted in the absence of any commercial or financial relationships that could be construed as a potential conflict of interest.

## Footnotes

1 https://www.genomaces.com/

2 https://www.chocogen.com/

3 https://www.pharmgkb.org/ accessed April 2018

4 https://www.coriell.org/0/Sections/Collections/NHGRI/1000Clm.aspx

5 https://www.chocogen.com/

6 https://www.pharmgkb.org/
