# Supplementary material for: Population Pharmacogenomics for Precision Public Health in Colombia
Source: Front Genet. 2019 Mar 22;10:241. doi: 10.3389/fgene.2019.00241 (PMC6439339; doi:10.3389/fgene.2019.00241)
Supplement: Supplementary file 2 [file Data_Sheet_1.PDF]

Supplementary Material

Population pharmacogenomics for precision public health in Colombia

Shashwat D. Nagar, A. Melissa Moreno, Emily T. Norris, Lavanya Rishishwar, Andrew B. Conley, Kelly L. O'Neal, Sara Vélez-Gomez, Camila Montes-Rodríguez, Wendy V. Jaraba-Álvarez, Isaura Torres, Miguel A. Medina-Rivas, Augusto Valderrama-Aguirre, I. King Jordan, and Juan Esteban Gallo

1 Supplementary Figures

Supplementary Figure 1. Ancestry associations for pharmaSNPs in Colombia. 2-3

Supplementary Figure 2. Comparison of the allele-specific PCR pharmaSNP genotyping assay results and the exome sequencing results. 4-6

Supplementary Figure 1. **Ancestry associations for pharmaSNPs in Colombia.** For each panel in the figure, pharmaSNP genotype percentages are shown for Antioquia (green) and Chocó (purple) followed by the ancestry association plots. For each genetic ancestry component – African (blue), European (orange), and Native American (red) – individuals' ancestry fractions (y-axis) are regressed against their pharmaSNP genotypes (x-axis). Ancestry associations are quantified by the slope of the regression (β) and its significance level (P).

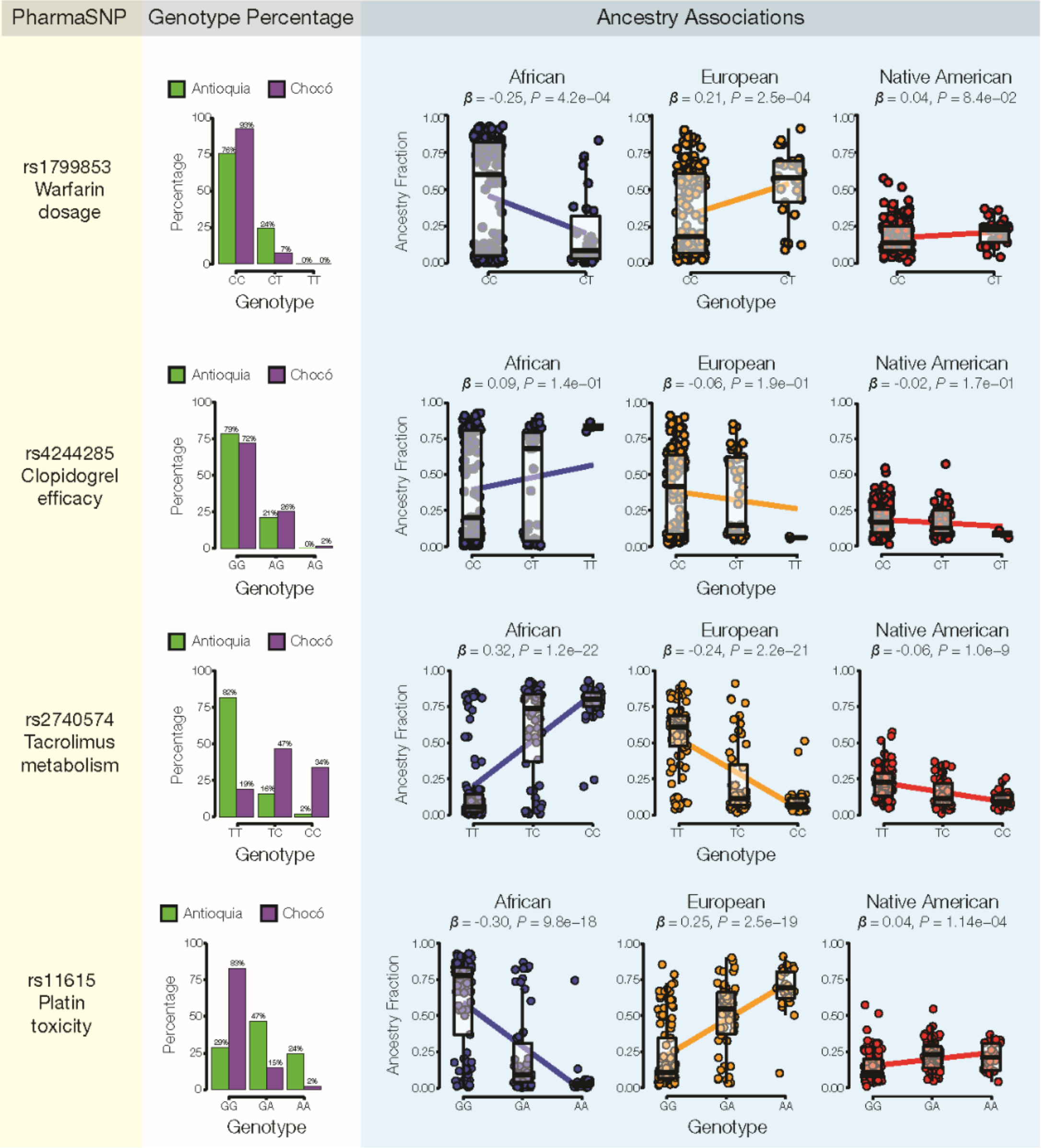

Supplementary Figure 1. **Ancestry associations for pharmaSNPs in Colombia.** [Continued from page 2].

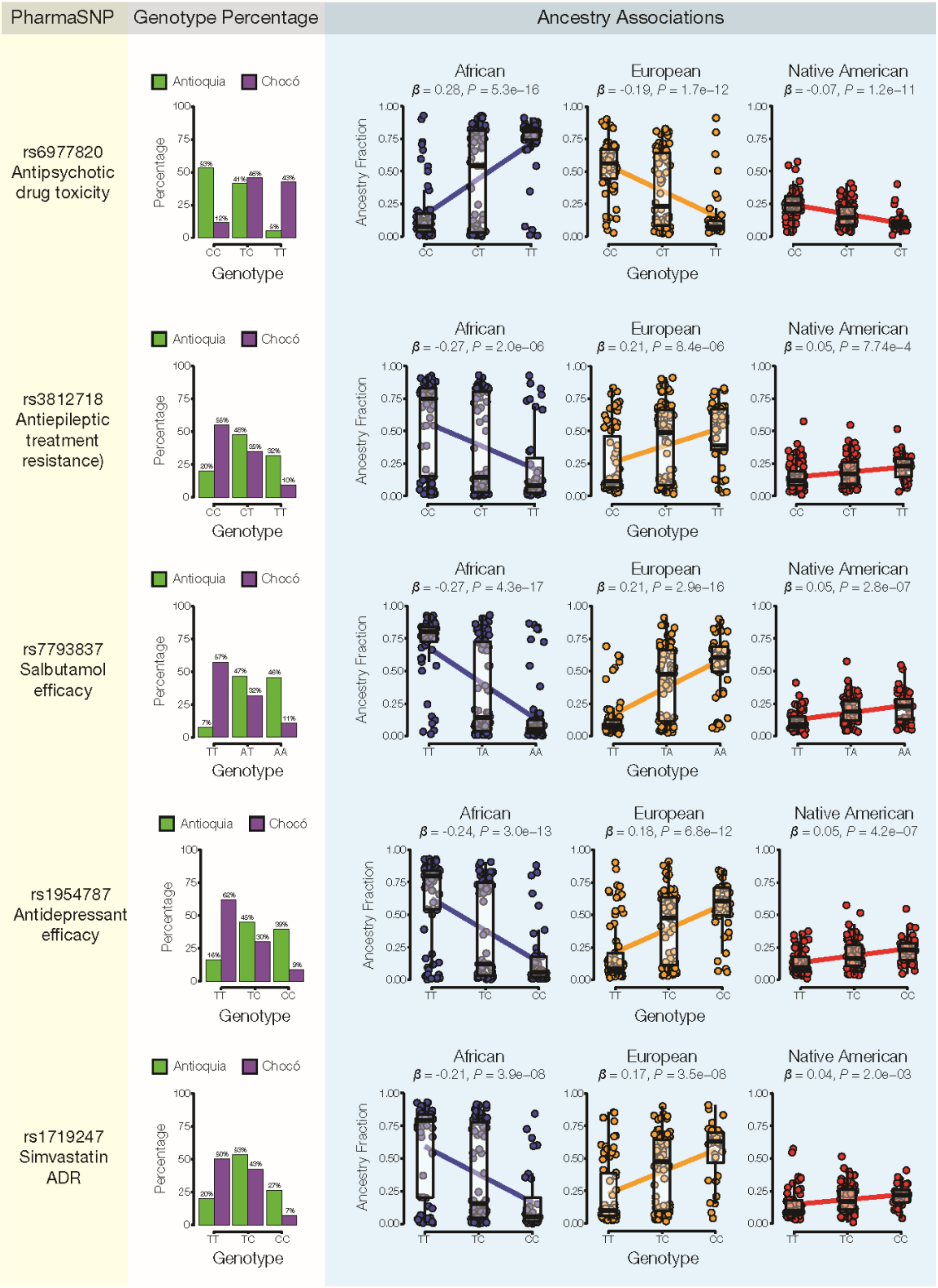
PharmaSNP
Genotype Percentage
Ancestry Associations
rs6977820 Antipsychotic drug toxicity
rs3812718 Antiepileptic treatment resistance)
rs7793837 Salbutamol efficacy
rs1954787 Antidepressant efficacy
rs1719247 Simvastatin ADR
Antioquia
Chocó
Percentage
Genotype
Ancestry Fraction
African
European
Native American
β = 0.28, P = 5.3e−16
β = -0.19, P = 1.7e−12
β = -0.07, P = 1.2e−11
β = -0.27, P = 2.0e−06
β = 0.21, P = 8.4e−06
β = 0.05, P = 7.74e−4
β = -0.27, P = 4.3e−17
β = 0.21, P = 2.9e−16
β = 0.05, P = 2.8e−07
β = -0.24, P = 3.0e−13
β = 0.18, P = 6.8e−12
β = 0.05, P = 4.2e−07
β = -0.21, P = 3.9e−08
β = 0.17, P = 3.5e−08
β = 0.04, P = 2.0e−03

Supplementary Figure 2. **Comparison of the allele-specific PCR pharmaSNP genotyping assay results and the exome sequencing results.** Three pharmaSNPs were genotyped in a 132 patient cohort from the GenomaCES laboratory in Medellín, Colombia using the custom allele-specific PCR assay described in the manuscript. Each individual PCR assay was validated via comparison with the exome sequence analysis results for these same patients. Taking the exome sequences as the ground truth for the presence of the pharmaSNP alleles in these patients, PCR results were scored as true positives (*TP*), false positives (*FP*), false negatives (*FN*), and true negatives (*TN*), and the following metrics were computed to validate the PCR genotyping assays for each individual genotype assayed:

$$Sensitivity = TP/(TP + FN)$$

$$Specificity = TN/(TN + FP)$$

$$Positive\ predictive\ value\ (PPV) = TP/(TP + FP)$$

$$Negative\ predictive\ value\ (NPV) = TN/(TN + FN)$$

The 95% confidence intervals for these metrics were computed as:

$$x \pm 1.96 * \sqrt{(x * (1 - x))/n}$$

where *x* is the value of the metric and *n* is the total number of genotype assays conducted.

rs4149056 (SLCO1B1*5)

Homozygous Non-effect Allele TT

		Exome YES	Exome NO	
PCR	YES	83	0	83
PCR	NO	3	46	49
		86	46	132

Sensitivity	96.5	95% CI	(93.4, 99.6)
Specificity	100	95% CI	(100, 100)
PPV	100	95% CI	(100, 100)
NPV	93.9	95% CI	(89.8, 98.0)

Heterozygous TC

		Exome YES	Exome NO	
PCR	YES	44	1	45
PCR	NO	0	87	87
		44	88	132

Sensitivity	100	95% CI	(100, 100)
Specificity	98.9	95% CI	(97.1, 100)
PPV	97.8	95% CI	(95.3, 100)
NPV	100	95% CI	(100, 100)

Homozygous Effect Allele CC

		Exome YES	Exome NO	
PCR	YES	2	2	4
PCR	NO	0	128	128
		2	130	132

Sensitivity	100	95% CI	(100, 100)
Specificity	98.5	95% CI	(96.4, 100)
PPV	50	95% CI	(41.5, 58.5)
NPV	100	95% CI	(100, 100)

rs1799583 (CYP2C9*2)

Homozygous Non-effect Allele CC

		Exome YES	Exome NO	
PCR	YES	113	1	114
PCR	NO	1	17	18
		114	18	132

Sensitivity	99.1	95% CI	(97.5, 100)
Specificity	94.4	95% CI	(90.5, 98.4)
PPV	99.1	95% CI	(97.5, 100)
NPV	94.4	95% CI	(90.5, 98.4)

Heterozygous CT

		Exome YES	Exome NO	
PCR	YES	17	1	18
PCR	NO	1	113	114
		18	114	132

Sensitivity	94.4	95% CI	(90.5, 98.4)
Specificity	99.1	95% CI	(97.5, 100)
PPV	94.4	95% CI	(90.5, 98.4)
NPV	99.1	95% CI	(97.5, 100)

rs1057910 (CYP2C9*3)

Homozygous Non-Effect Allele AA

		Exome YES	Exome NO	
PCR	YES	115	0	115
	NO	0	17	17
		115	17	132

Sensitivity	100	95% CI	(100, 100)
Specificity	100	95% CI	(100, 100)
PPV	100	95% CI	(100, 100)
NPV	100	95% CI	(100, 100)

Heterozygous AC

		Exome YES	Exome NO	
PCR	YES	17	0	17
	NO	0	115	115
		17	115	132

Sensitivity	100	95% CI	(100, 100)
Specificity	100	95% CI	(100, 100)
PPV	100	95% CI	(100, 100)
NPV	100	95% CI	(100, 100)
